# Alterations in oral bacterial communities are associated with risk factors for oral and oropharyngeal cancer

**DOI:** 10.1038/s41598-017-17795-z

**Published:** 2017-12-15

**Authors:** Daniela Börnigen, Boyu Ren, Robert Pickard, Jingfeng Li, Enver Ozer, Erica M. Hartmann, Weihong Xiao, Timothy Tickle, Jennifer Rider, Dirk Gevers, Eric A. Franzosa, Mary Ellen Davey, Maura L. Gillison, Curtis Huttenhower

**Affiliations:** 1 Department of Biostatistics, Harvard T.H. Chan School of Public Health, Harvard University, Boston, MA 02115 USA; 2 The Broad Institute of MIT and Harvard, Cambridge, MA 02115 USA; 3 The Ohio State University Comprehensive Cancer Center, Columbus, OH 43202 USA; 4 Biology and the Built Environment Center and Institute of Ecology and Evolution, University of Oregon, Eugene, OR 97403 USA; 5 Present Address: Department of Civil and Environmental Engineering, Northwestern University, Evanston, IL 60208 USA; 6 Department of Epidemiology, Harvard T.H. Chan School of Public Health, Harvard University, Boston, MA 02115 USA; 7 Department of Oral Biology, College of Dentistry, University of Florida, Gainesville, FL 32610 USA; 8 Present Address: University Heart Center Hamburg-Eppendorf, Clinic for General and Interventional Cardiology, Hamburg, Germany; 9 German Center for Cardiovascular Research (DZHK), Hamburg/Lübeck/Kiel Partner Site, Hamburg, Germany

## Abstract

Oral squamous cell carcinomas are a major cause of morbidity and mortality, and tobacco usage, alcohol consumption, and poor oral hygiene are established risk factors. To date, no large-scale case-control studies have considered the effects of these risk factors on the composition of the oral microbiome, nor microbial community associations with oral cancer. We compared the composition, diversity, and function of the oral microbiomes of 121 oral cancer patients to 242 age- and gender-matched controls using a metagenomic multivariate analysis pipeline. Significant shifts in composition and function of the oral microbiome were observed with poor oral hygiene, tobacco smoking, and oral cancer. Specifically, we observed dramatically altered community composition and function after tooth loss, with smaller alterations in current tobacco smokers, increased production of antioxidants in individuals with periodontitis, and significantly decreased glutamate metabolism metal transport in oral cancer patients. Although the alterations in the oral microbiome of oral cancer patients were significant, they were of substantially lower effect size relative to microbiome shifts after tooth loss. Alterations following tooth loss, itself a major risk factor for oral cancer, are likely a result of severe ecological disruption due to habitat loss but may also contribute to the development of the disease.

## Introduction

Head and neck squamous cell carcinomas are a major cause of cancer morbidity and mortality, with an estimated incidence of 549,000 cases worldwide in 2008^1^. The majority of these are oral cancers arising in the oral cavity and oropharynx, for which tobacco usage, betel chewing, alcohol consumption, and human papillomavirus (HPV) infection are established risk factors^2–9^. Case-control studies have also reported associations between oral cancer and measures of chronic poor oral hygiene (e.g. loose or missing teeth, infrequent tooth brushing or dental visits)^5,10–20^, even among non-smokers and non-drinkers^21,22^. When combined with tobacco or alcohol use, poor oral hygiene acts synergistically to increase the risk associated with either exposure alone^21,22^ and leads to chronic infection and inflammation, both of which are increasingly recognized in the pathogenesis of cancer^23–25^ and as factors in carcinogenic feedback loops incorporating the resident microbiota. Although a few recent studies characterized the interactions of these epidemiological risk factors for oral cancer with the microbiome, they were limited by sample size and different experimental approaches^26–29^.

Apropos, human microbiome studies have recently characterized the structure and function of the microbial communities in different regions of the human body during health^30^ and disease states, including the oral cavity^31–35^. Indeed, specific microbial communities are associated with periodontitis^36–38^ and dental caries^39–41^. Preliminary small studies have also found different microbial communities in samples collected from the surface of oral cancers and normal tissues matched from the same subject^42–44^. A sample of 15 oral cancers, for example, were enriched for Firmicutes and Actinobacteria relative to matched samples^45^ and a single array-based case-control study reported elevated counts of a few bacteria in oral cancer (e.g. *Capnocytophaga gingivalis*, *Provatella melaningtoenica*, and *Streptococcus mitis*)^46^. Larger-scale epidemiologically designed cohorts are needed to verify these structural associations, however, and especially to provide well-powered investigations of host-microbe-environment interactions in oral cancer.

In this study, we thus compared the oral microbiome in 121 oral cancer cases and 242 matched controls using multivariate analysis targeted at microbial taxonomic and inferred functional profiles. We evaluated associations between lifestyle factors (alcohol and tobacco use), health characteristics (history of periodontitis, tooth status), case-control status, and the composition and function of oral microbial communities (Table 1). Our results revealed strong shifts in composition and function associated with tooth status, with more modest shifts during tobacco smoking and oral cancer, both while additionally incorporating environmental factors into the analysis. Microbial community effects included a loss of ecological diversity and increased glutamate metabolism in tobacco smoking individuals, disruptions in both structure and function in individuals after tooth loss, and a decrease in glutamate metabolism and metal transport in cancer cases. Other lifestyle and health characteristics, including alcohol consumption, history of periodontitis, and tumor HPV status, were associated only with minor microbial effects in this population. We conclude that alterations in the structure, diversity, and function of the oral microbiome occur in association with established risk factors for oral cancer, especially after complete tooth loss; these alterations may contribute to disease development but are likely due to severe ecological disruption and habitat loss.

**Table 1:** Cohort characteristics including case-control status, lifestyle, and health. *P*-values are two-tailed and correspond to Fisher’s exact tests for differential enrichment of categorical properties in cases relative to controls. As expected, most risk factors are significantly enriched in cancer cases.

		Total	Case (N)	Case (%)	Control (N)	Control (%)	*P*
*Cohort description*		363	121		242		
*Demographic characteristics*							
**Gender**							
Female		81	27	22.3	54	22.3	1.000
Male		282	94	77.7	188	77.7	1.000
*Lifestyle Characteristics*							
**Current/former cigarette usage**	**# of cigarettes**						
Never		103	22	18.2	81	33.5	0.003
Slight former user		46	6	5.0	40	16.5	0.001
Never regular user		13	4	3.3	9	3.7	1.000
Former regular user		141	57	47.1	84	34.7	0.030
Current regular user	21/day (mean), 20/day (median)	60	32	26.4	28	11.6	<0.001
**Current/former alcohol usage**	**# of drinks/week**						
Never		8	5	4.1	3	1.2	0.123
Never regular or former regular		187	59	48.8	128	52.9	0.504
Current regular	22/week (mean), 8/week (median)	168	57	47.1	111	45.9	0.824
*Health characteristics*							
**Current tooth status**							
Has all or most of natural adult teeth		248	65	53.7	183	75.6	<0.001
Has partial plates or implants		37	11	9.1	26	10.7	0.715
Has full upper dentures or implants		21	10	8.3	11	4.5	0.160
Has full lower dentures or implants		1	0	0.0	1	0.4	1.000
Has upper and lower dentures		39	24	19.8	15	6.2	<0.001
NA		17	11	9.1	6	2.5	0.008
**Oral rinse sample HPV status**							
Negative		82	63	52.1	19	7.9	<0.001
Positive		44	43	35.5	1	0.4	<0.001
NA		237	15	12.4	222	91.7	<0.001
**Tumor HPV status**							
Negative	HPV cDNA <3 or RPLP0 is not valuable	13	13	10.7			
Positive	HPV cDNA ≥3 and RPLP0 is evaluable	21	21	17.4			
NA	Missing	329	87	71.9	242	100.0	
**History of periodontitis**							
Negative		269	80	66.1	189	78.1	0.016
Positive		82	35	28.9	47	19.4	0.046
NA		12	6	5.0	6	2.5	0.225
*Other information*							
**Sequencing phase**	**Plates**						
Phase1	1YXO, 1YXP, 1YXR, 1YXS	234	90	74.4	144	59.5	0.005
Phase2	SK-24Q7, SK-24Q8, IZGE	40	15	12.4	25	10.3	0.595
Phase3	SK-27BR	89	16	13.2	73	30.2	<0.001

## Results

### Study population and sample characteristics

Oral rinse samples were collected for microbiome analysis from 121 cases diagnosed with oral cavity (n = 43), oropharynx (n = 64), or unknown primary (n = 5) squamous cell carcinoma (Table 1). A total of 242 controls were age (5-year intervals) and gender matched to cases at a 2:1 ratio within six months of case enrollment. The cohort was predominantly male, with median age 58 years (IQR 53–66). Cases were more likely than controls to be current tobacco users or cigarette smokers. Complete tooth loss and a history of periodontitis were also more common among cases than controls.

### The structure and function of the oral microbiome is associated with oral cancer status

**Figure 1:**
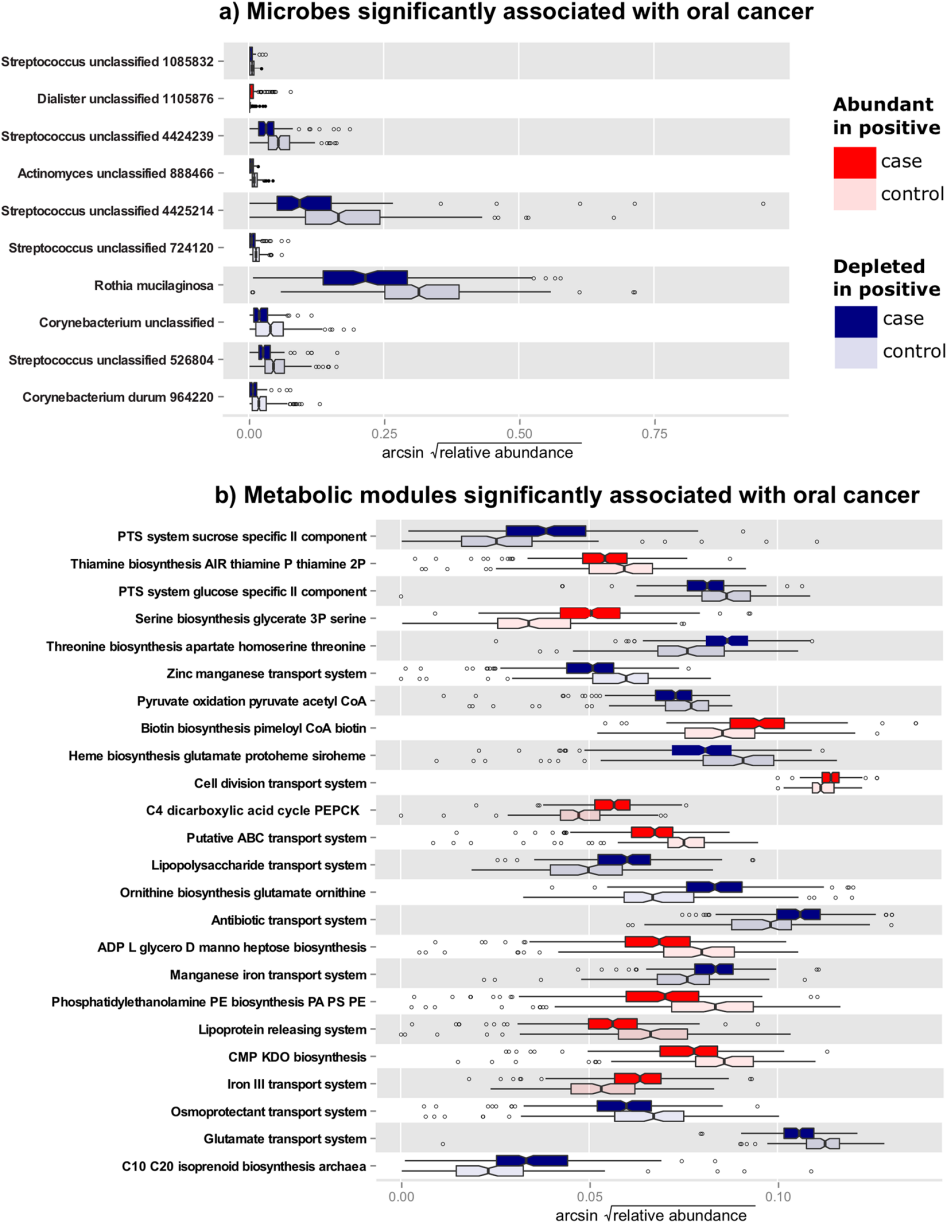
Oral microbial community taxa and functional pathways differentially abundant in cancer. (**a**) Taxa (genera and OTUs) and (**b**) pathways differentially abundant in oral cancer microbiomes as determined by a multivariate model incorporating case/control status, tumor HPV status, tooth loss, periodontal disease, and other demographic and clinical covariates (see Methods). Differences are significant at FDR *q* < 0.25, and *n* = 121 cases, 242 controls (see Table 1, Supplementary Datasets 4 and 5).

A comparison of the oral microbiome in cancer cases and controls showed changes both in structure (16 significantly differentially abundant clades) and in function (25 metabolic pathways, Supplementary Datasets 4 and 5 and Fig. 1). Tests were performed using a multivariate generalized linear model incorporating case/control status, tooth loss, tumor HPV status (for cases), periodontal disease, tobacco usage, alcohol consumption, and other covariates, with microbial abundances as outcome and FDR-corrected significance at *q* < 0.25; see Methods. Taxa belonging to the genus *Dialister* occurred at higher relative abundances in cases, whereas the orders Actinomycetales and Lactobacillales were significantly under-represented in oral cancer (Fig. 1a). No significant differences in microbial presence/absence were observed between cases and controls, in contrast to these changes in specific clades’ relative abundances.

Analysis of predicted functional modules indicated that genes involved in synthesis of lipopolysaccharides (KEGG^47^ M00063: CMP-KDO biosynthesis and M00064: ADP-L-glycero-D-manno-heptose biosynthesis) were more abundant in cases than in controls (Fig. 1b). Pathways involved in LPS transport (e.g. M00250: Lipopolysaccharide transport) decreased, possibly reflecting a shift to a higher proportion of a distinct group of Gram-negative bacteria, e.g. a shift to higher levels of *Dialister*. Other functional modules, in particular the relative abundance of genes involved in amino acid transport and synthesis were altered; specifically an increase in serine synthesis (M00020: Serine biosynthesis, glycerate-3P => serine) was detected, while glutamate uptake (M00233: Glutamate transport), synthesis of threonine from aspartate (M00018: Threonine biosynthesis, aspartate => homoserine => threonine), and synthesis of ornithine from glutamate (M00028: Ornithine biosynthesis, glutamate => ornithine) were at a lower relative abundance in oral cancer subjects. As these pathways are generally involved in oxidative energy harvest from TCA cycle products, particularly in combination with reduced LPS transport, this may reflect a shift toward anaerobic microbes and/or metabolism in the oral cancer microenvironment.

Community functional dysbioses during oral cancer were also reflected in a predicted increase in the synthesis and transport of vitamins and cofactors involved in metabolism, e.g. biotin and thiamine (M00123: Biotin biosynthesis, pimeloyl-ACP/CoA => biotin, M00127: Thiamine biosynthesis, AIR => thiamine-P/thiamine-2P). There was also a shift in iron uptake systems (M00190: Iron(III) transport, M00243: Manganese/iron transport), iron (M00243: Manganese/iron transport, M00248: ABC transporter), zinc, and manganese transport (M00244: Putative zinc/manganese transport). This suite of depleted metal transporters, primarily iron, may also reflect the need for fewer of these cofactors in a less-oxidative metabolic environment. Lastly, a decrease in transport systems for osmoprotectants (M00209: Osmoprotectant transport) was predicted, which may reflect a community response to changes in the osmolarity of saliva. Epithelial cell death and the resultant release of cell constituents, including amino acids, may be higher in cancer patients, hence synthesis of osmoprotectants may not be necessary.

### Lower microbial diversity and loss of function after tooth loss

**Figure 2:**
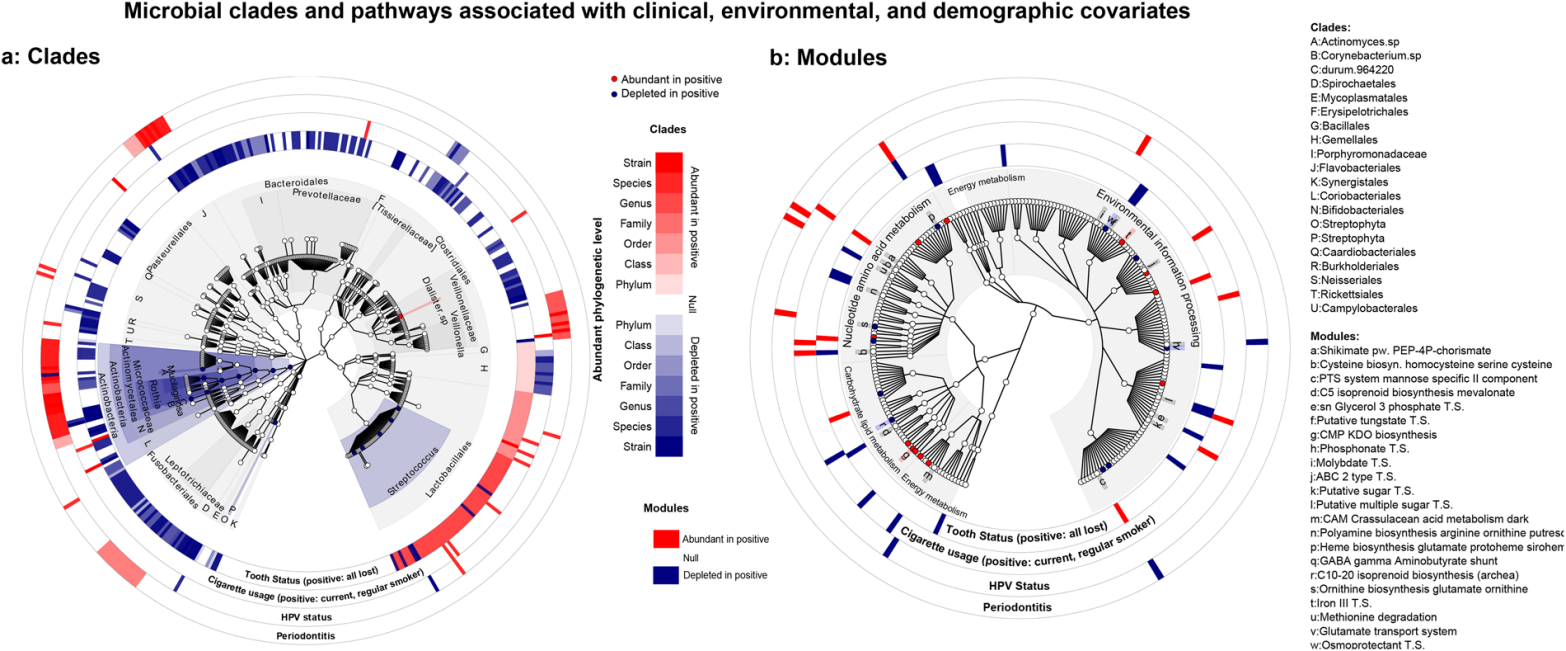
Microbial clades and pathways associated with oral cancer and with clinical, environmental, and demographic covariates. (**a**) Clades (tree includes all microbes in the dataset) and (**b**) pathways (shown on the KEGG BRITE hierarchy) differentially abundant with respect to oral cancer (highlighted points) and a subset of non-cancer covariates in this cohort (outer bands), comprising periodontal health, tumor HPV status, tobacco usage, and tooth status; multivariate model is as in Fig. 1 and Methods. Highlighted associations are significant at FDR *q* < 0.25, *n* as in Table 1.

**Figure 3:**
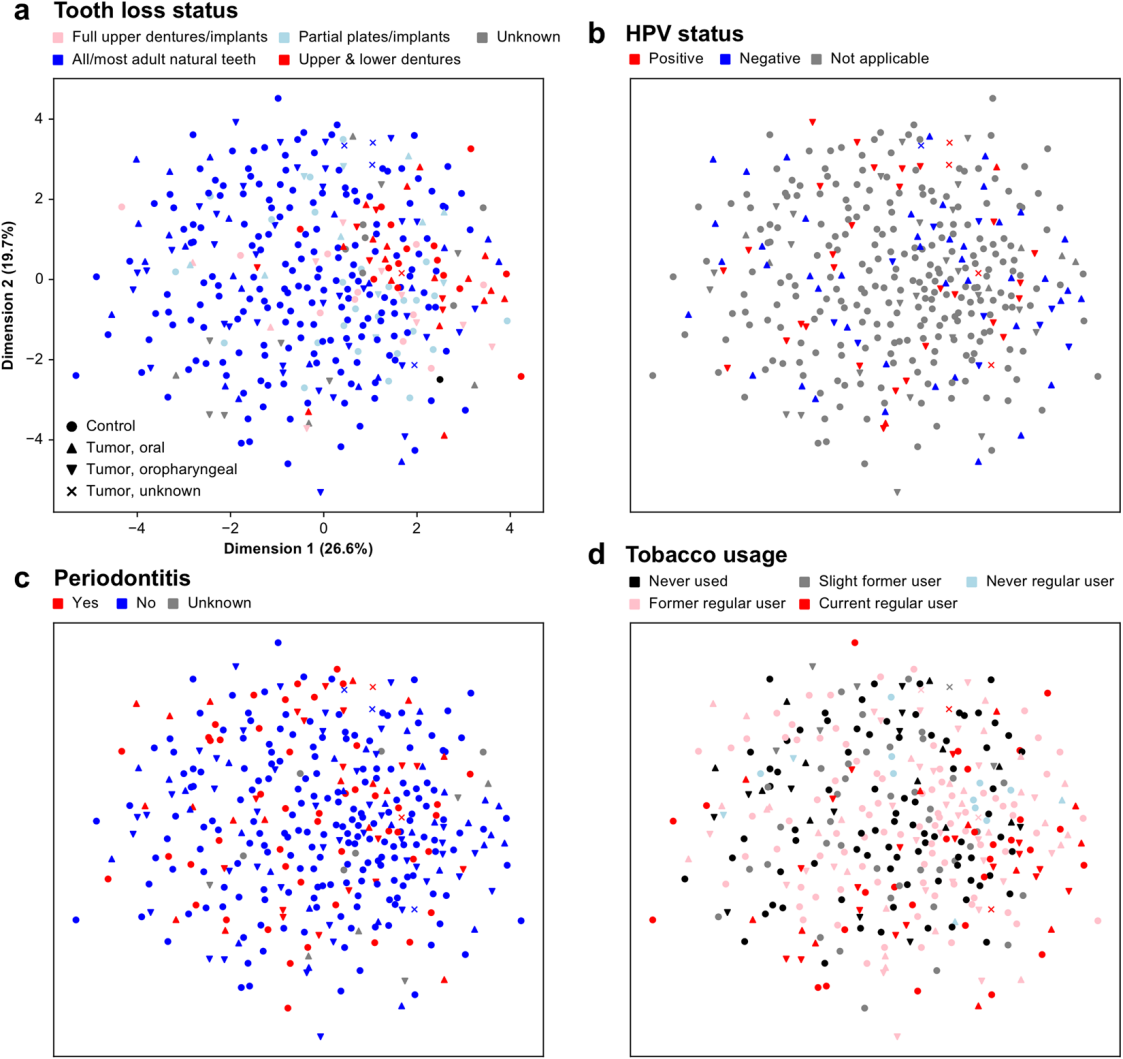
Covariation of microbial community beta-diversity with non-cancer covariates including tooth loss, periodontal health, tumor HPV status, and tobacco usage. Ordination by non-parametric multidimensional scaling of samples’ Canberra dissimilarities, with oral/oropharyngeal cancer status indicated by shape and color stratified by (**a**) tooth loss status, (**b**) HPV positivity, (**c**) periodontal health, and (**d**) tobacco usage. Complete tooth loss represents the largest determinant of variability in the cohort’s oral microbial communities, with smaller effects of cancer case/control status and other covariates.

**Figure 4:**
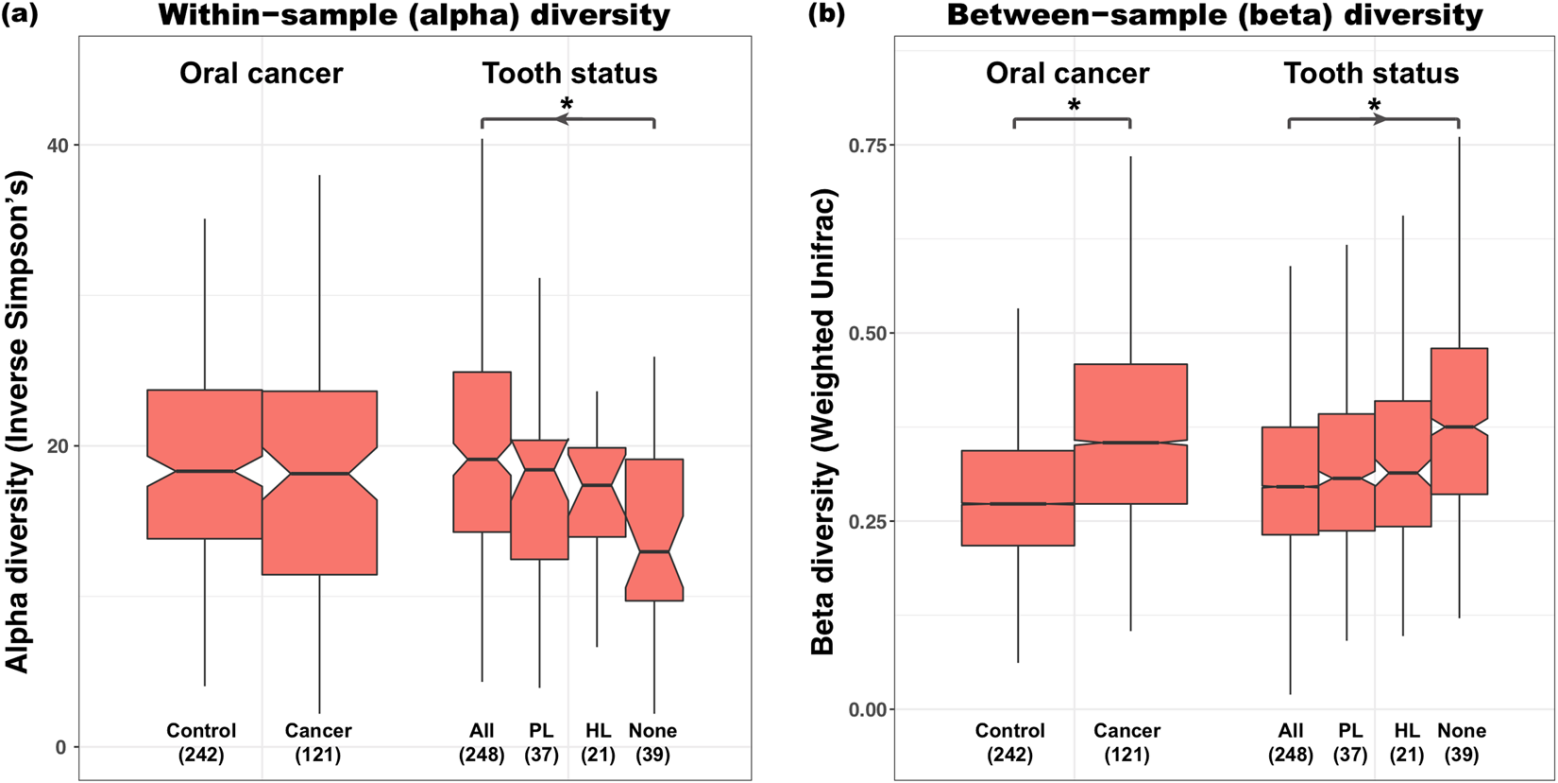
Oral cancer and tooth loss significantly affect microbial community alpha- and beta-diversity. (**a**) Within-sample inverse Simpson alpha-diversity across all samples, and (**b**) between-sample Bray-Curtis dissimilarity between all pairs of samples within each phenotype. Stars indicate significant differences by Wilcoxon rank sum test (binary oral cancer status) or for Cuzick’s trend test (ordinal tooth loss status), both at *p* < 0.05.

We observed a dramatic decrease in a broad range of clades in association with total tooth loss (i.e. no natural teeth remaining), comprising 122 clades (from Actinomycetaceae, Corynebacterium, Rothia, Prevotella, Flavobacteriales, Streptococcaceae, Fusobacteriales, and Proteobacteria; see Supplementary Dataset 4, Fig. 2). This represented the largest single association of the microbiome with clinical, demographic, or environmental factors in this cohort (Fig. 3). The striking difference between even one versus no natural teeth remaining likely corresponds to a loss of habitat, since the epithelium-tooth interface is a key site for bacterial colonization in the oral cavity. Individuals with total tooth loss had less diverse oral microbiomes that were less similar to each other than in individuals with good oral hygiene, revealing a significant microbial community profile shift associated with complete tooth loss (Fig. 4).

**Figure 5:**
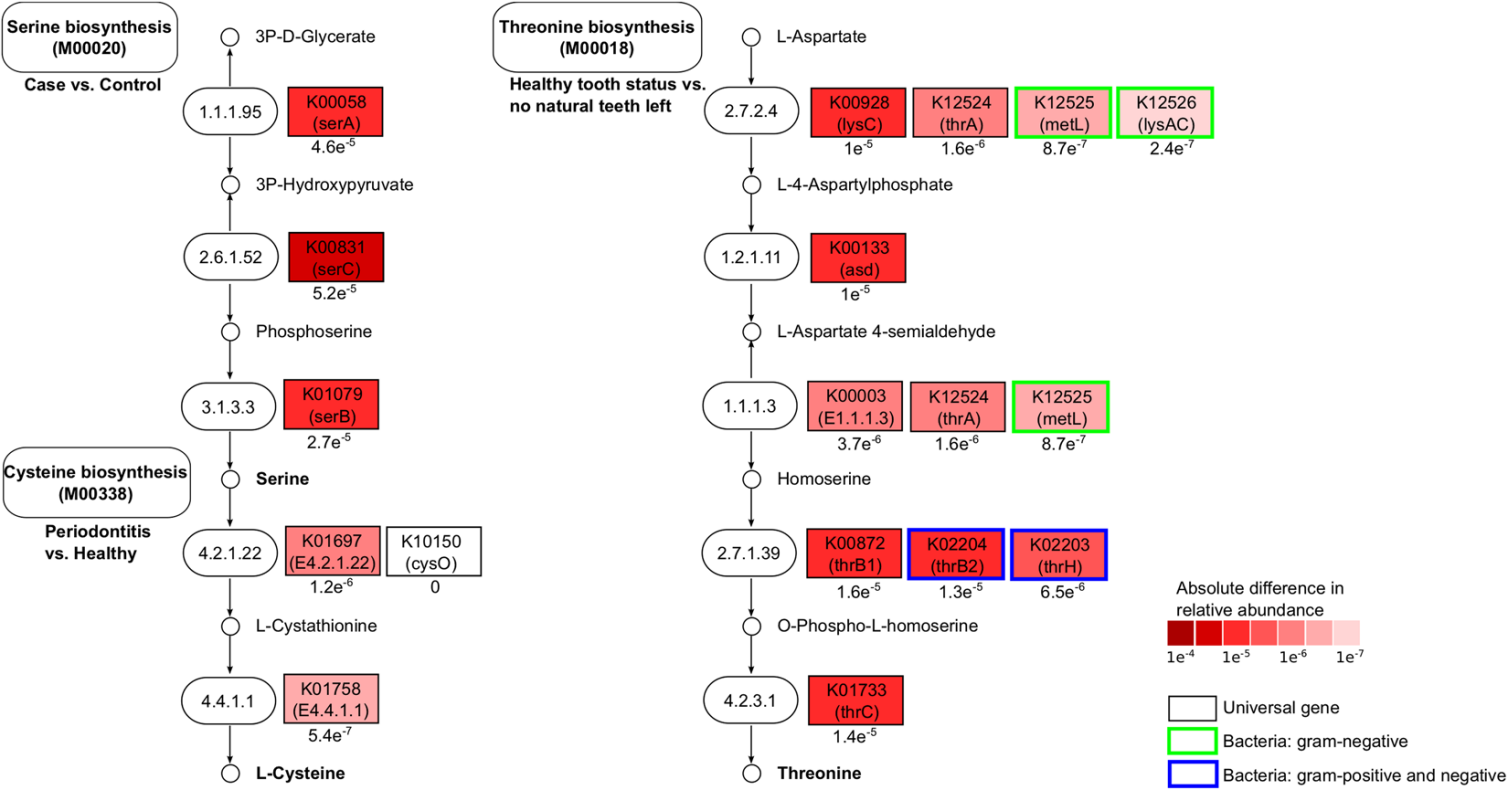
Gene-level analysis of the serine biosynthesis, cysteine biosynthesis, and threonine biosynthesis pathways. The results of our study showed significant changes in the serine biosynthesis pathway (M00020) in cancer patients as compared with healthy individuals, cysteine biosynthesis pathway (M00338) in periodontitis patients, and the threonine biosynthesis pathway (M00018) in patients with complete tooth loss. Here, these pathways are shown on a gene level by visualizing the absolute difference in relative abundances of the genes (red color scheme), revealing that most genes from these pathways have been altered in their abundance between both conditions on a gene level. Additionally, we demonstrate that some of the genes from the threonine biosynthesis are either specific for Gram-negative (green) or Gram-negative/-positive bacteria (blue), while the serine and cysteine biosynthesis pathways consists of universal genes present in eukaryotes and prokaryotes.

The broad shift in community composition was also reflected in a dramatic shift in function associated with tooth loss. Twenty-two metabolic pathways (Supplementary Dataset 5), including transport systems for sugars (M00215: D-Xylose transport, M00216: Multiple sugar transport, M00217: D-Allose transport, M00198: Putative sn-glycerol-phosphate transport), metals (M00245: Cobalt/nickel transport, M00186: Tungstate transport), and organic ions, such as glycine, betaine, and proline (M00208: Glycine betaine/proline transport), as well as modules involved in the synthesis of polyamines, including arginine, agmatine, ornithine, putrescine, and spermidine (M00133: Polyamine biosynthesis, arginine => agmatine => putrescine => spermidine, M00134: Polyamine biosynthesis, arginine => ornithine => putrescine) were lower in abundance in individuals with tooth loss. Other modules at lower abundance included those for the GABA gamma aminobutyrate shunt, which channels glutamate into the TCA cycle (M00027: GABA (gamma-Aminobutyrate) shunt). In contrast, many pathways including the synthesis of threonine, asparate, and homoserine (M00018: Threonine biosynthesis, aspartate => homoserine => threonine) and phosphoribosyl pyrophosphate (M00005: PRPP biosynthesis, ribose 5P => PRPP), which is involved in the synthesis of nucleotides (purines and pyrimidines), NAD, and the amino acids histidine and tryptophan, occurred at higher abundance among subjects with no remaining natural teeth. A gene level analysis of the Threonine biosynthesis pathway (M00018) revealed that most genes were altered in their relative abundance between individuals having at least some of their natural teeth and individuals with complete tooth loss (Fig. 5). Additionally, some of the genes from this pathway, e.g. K12525 (metL), K12526 (lysAC), which are interchangeable within the process from L-Aspartate into L-4-Aspartylphosphate are specific to Gram-negative bacteria. Overall, complete tooth loss results in a significant change in the structure and function of the oral microbiome.

### Tobacco smoking leads to a loss of diversity and increased glutamate metabolism in the oral microbiome

To analyze the influence of tobacco smoking on the oral microbiome, we identified clades and metabolic pathways that differed significantly in current regular smokers (60 individuals) and occasional or former smokers (200) compared to non-smokers (103; Table 1). This revealed 24 clades (Fig. 2a) and 12 metabolic pathways (Fig. 2b) that were altered in smokers compared to non-smokers (Supplementary Datasets 4 and 5) and an increased between-sample microbial diversity in smokers (Supplementary Fig. 1). Past smoking habits did not have a significant effect on the microbial community profile for most clades and pathways.

Most of the significant differences in clades were between regular smokers and non-smokers (24 clades). Comparing these groups, a broad range of genus-level clades were significantly increased in smokers, including *Lactobacillus*, *Bifidobacterium*, *Atopobium*, *Prevotella*, *Streptococcus*, and *Veillonella*. On the other hand, *Rothia*, *Neisseria*, and *Lautropia* were significantly decreased in regular smokers. Shifts observed in a few significant clades (5) among occasional or former smokers were concordant with the differences detected between regular smokers and non-smokers. One proposed explanation for these observations is that smoking disrupts niche saturation of health-associated species, eliminating a subset of organisms, altering community composition, lowering alpha-diversity, and increasing beta-diversity. In line with this model, our data show an overall increased between-sample microbial diversity in smokers as compared to non-smokers (Supplementary Fig. 1).

Significant changes in functional modules were detected in smokers, specifically an increased abundance of transport systems, including sugar uptake (M00197: Putative fructooligosaccharide transport, M00216: Multiple sugar transport), phosphate uptake (M00222: Phosphate transport) and metal transport systems, such as manganese, zinc, and iron (M00319: Manganese/zinc/iron transport). An increase in abundance of the GABA gamma aminobutyrate shunt was also detected again (M00027: GABA (gamma-Aminobutyrate) shunt), used by a variety of bacteria, in particular species of *Streptococcus* and *Lactobacillus* ^48,49^, to cope with acid and oxidative stress^50^. Likewise, genes involved in amino acid degradation, specifically the conversion of histidine into glutamate, were also increased (M00045: Histidine degradation, histidine => N-formiminoglutamate => glutamate), indicating that pathways involved in glutamate metabolism are represented at higher levels in patients that smoke on a regular basis.

### Limited structural changes and moderate functional implications tied to tumor HPV status, periodontitis, and alcohol consumption

Our multivariate model of cohort features associated with the microbiome included several other clinical and environmental factors (see Methods), but none of these showed evidence of associations as strong as those above for oral cancer or tooth loss (Table 1, Fig. 2). First, we assessed differences in microbial composition and function between patients with HPV-positive (35) and HPV-negative (55) oral cancer. Members of the *Actinomyces*, *Granulicatella*, *Oribacterium*, and *Campylobacter* genera as well as *Veillonella dispar*, *Rothia mucilaginosa*, and *Haemophilus parainfluenzae* were significantly increased in patients with HPV-positive cancers compared to non-HPV cancer patients.

In contrast, *Streptococcus anginosus*, *Peptoniphilus*, and *Mycoplasma* were significantly decreased in HPV-positive cancers. Functional changes in the oral microbial communities of HPV-positive vs. HPV-negative cancer patients included phosphonate transport (M00223: Phosphonate transport) and amino acid metabolism through heme (M00121: Heme biosynthesis, glutamate => protoheme/siroheme), Shikimate (M00022: Shikimate pathway, phosphoenolpyruvate + erythrose-4P => chorismate), glutamate, and ornithine (M00028: Ornithine biosynthesis, glutamate => ornithine, Supplementary Dataset 5). We further investigated stratification of the oral cancer cases by site (oral vs. oropharyngeal) as well as HPV status, yielding 52 oral cancers of which 2 (4%) were positive for HPV, 44 (85%) negative, and 6 (11%) unspecified; and 64 oropharyngeal cancers of which 29 (45%) were HPV-positive, 10 (16%) negative, and 25 (39%) unspecified (5 cancer cases with unspecified sites were excluded from this analysis). Likely due to the small numbers resulting from this stratification, we did not identify any oral-oropharyngeal differences that were statistically significant after multiple hypothesis testing correction (Mann-Whitney U FDR *q*-value < 0.1). The same was true when stratifying the analysis by tumor HPV status.

Few significantly different clades were detected in patients with a history of periodontal disease (82 subjects) as compared to those without (269 subjects, Supplementary Dataset 4); only the *Fusobacterium*, *Leptotrichiaceae*, *Eikenella*, and *Capnocytophaga* genera were increased. Importantly, these are all Gram-negative bacteria, in line with previous findings that periodontitis is characterized by a shift from primarily Gram-positive to Gram-negative organisms^51^. Likewise, at the functional level, few modules were consistently different (Supplementary Dataset 5). Genes for mannose uptake (M00276: PTS system, mannose-specific II component) were less abundant; while subtle, this likely reflects the shift from a predominately Gram-positive community to Gram-negative, as sugar transport and metabolism is primarily attributed to the early tooth-colonizing Gram-positive streptococci. Interestingly, the higher abundance of Gram-negative anaerobes was associated with an increase in L-cysteine synthesis, both directly and indirectly through degradation of methionine (M00338: Cysteine biosynthesis, homocysteine + serine => cysteine, M00035: Methionine degradation). In addition to being an amino acid for protein synthesis, L-cysteine acts as an antioxidant and is also metabolized to hydrogen sulfide (H_2_S)^52^.

Finally, only one clade was significantly linked to alcohol consumption when comparing microbial profiles among current regular alcohol drinkers (168), former or irregular drinkers (187), and subjects never drinking alcohol (8, Supplementary Dataset 4). This represented an unclassified species in genus *Capnocytophaga* marginally decreased in current regular drinkers. No changes in metabolic modules were associated with alcohol consumption status, and even in targeted tests of only heavy (≥3 drinks per day) and non-drinkers, only two clades were enriched (*Sharpea* and unclassified Veillonellaceae) and one depleted (*Lactococcus*, Mann-Whitney U FDR *q* < 0.1). Notably, these were all relatively minor genera across the samples, and none of the differences in group means exceeded 0.1% relative abundance.

## Discussion

In this study, we assessed structural and functional changes in the oral microbiome during oral cancer, in tandem with clinical, demographic, and environmental covariates. This multivariate analysis remained well-powered compared to previous studies^42,43^ due to our sample size of over 120 oral cancer cases and twice as many matched controls. Few specific genera were consistently enriched or depleted during cancer, while functional shifts included decreased potential for microbial glutamate metabolism and metal (particularly iron) transport. The greatest dysbioses arose during tooth loss, particularly when no natural teeth remained, and other oral cancer risk factors including tobacco smoking, HPV infection, and periodontitis showed significant but smaller effects (Supplementary Dataset 4 and 8).

A small number of genera were differentially abundant during oral cancer, specifically *Dialister* (enriched) and *Scardovia* (depleted). *Dialister* are Gram-negative anaerobes within the family Veillonellaceae that have been associated with worse periodontal status^53^ and endodontic infections^54,55^. They also appear to compete with species of *Scardovia*, as co-occurence analysis determined that these two genera infrequently inhabit the same niche^56^. Although previous sample sizes have been limited, earlier culture-based and culture-independent studies have also reported on oral bacteria disrupted on or in oral and esophageal tumors or in associated saliva samples^42,43,46,57–61^. In one study by Hooper *et al*.^61^, viable bacteria were detected within carcinoma tissues themselves. Our results generally agree with the enriched microbes, as well as those published by Pushalker *et al*., where bacterial colonization of tumor tissue and normal mucosa from the same subject were again found to be distinct^43^.

Previous studies suggested that poor oral hygiene could, in addition to increasing the risk of oral cancer^16^, influence the oral microbiome in cancer patients. The largest microbial effect in our cohort occurred during complete tooth loss, with progressive dysbioses with partial loss. The accompanying loss of community diversity was caused by the depletion of many clades known to inhabit a variety of distinct environments around the teeth (e.g. enamel, epithelial interface, or sub-gingival crevice), reducing Actinomycetaceae, *Corynebacterium*, *Rothia*, *Prevotella*, Flavobacteriales, Streptococcaceae, Fusobacteriales, and Proteobacteria and leaving a community of primarily Lactobacillales. Given the loss of habitats, the depletion of clades ranging up to the phylum level is unsurprising, as is the corresponding loss in inferred metagenomic functional potential.

Tobacco smoking also negatively influences oral health^62,63^ and can lead to oral cancer^21,22,64^. In particular, smoking can affect microbial biofilm structure and can result in unstable colonization compared to non-smokers^62^, increasing susceptibility to bacterial infections in smokers by dysregulation of the innate and adaptive immune responses^63^. Here, although microbial diversity changes associated with smokers were not statistically significant, we observed a trend toward increased overall between-sample diversity in smokers (Supplementary Fig. 1). This is in line with a previous study showing divergent microbial colonization patterns in smokers^62^. We likewise observed altered abundances of Firmicutes (*Lactobacillus*, *Veillonella*, *Streptococcus*), Actinobacteria (*Bifidobacterium*, *Atopobium*), Proteobacteria (*Neisseria*), and Bacteroidetes (*Prevotella*), also in agreement with previous findings^26,62,65,66^. Smoking may thus lead to a higher abundance of “misplaced” indigenous bacteria: depletion of niches typically colonized by commensal microbes and a greater incidence of atypical organisms, preventing stable biofilm formation and immune development.

HPV is similarly responsible for a large variety of cancer types^67–70^, including head and neck squamous cell carcinomas^71^. Previous studies have reported *Streptococcus* spp. as cofactors in the malignant transformation of oral keratinocytes by HPV^72–75^, although we detected relatively weak associations specific to HPV-positive oral cancers. Six metabolic pathways were significantly different, in addition to several clades including decreased *Streptococcus anginous* and *Mycoplasma* and increased *Veillonella dispar*. Previous studies have also suggested that *Mycoplasma* infection increases the rate of HPV infection, as well as the risk for abnormal cervical cytology and cancer^76,77^. Finally, glutamate metabolism and metal transport were, in contrast to other cancers, significantly increased in patients with HPV-positive tumors (as well as during several other indicators of poor oral health), in line with findings that report transglutaminase 2 (and other enzymes) as inhibitors of HPV^78^ or modulators of viral infections^79,80^.

Finally, periodontitis is an inflammatory condition resulting from a combination of microbial challenge and disregulated host immune response^81^. It is tightly linked to a shift to a higher proportion of Gram-negative anaerobes^36–38^ and is causally associated with oral cancer. In this study, four genus-level clades were significantly increased in individuals with periodontitis, namely *Fusobacterium*, *Leptotrichiaceae*, *Eikenella*, and *Capnocytophaga*. We detected a significant increase in abundance of the Gram-negative anaerobe *Fusobacterium*, in agreement with previous findings^82^. In fact, the ability of *Fusobacterium* to manipulate the immune response and support the growth and colonization of both *Eikenella* and *Capnocytophaga* species (the other genera found at higher abundance) is well documented^83^, which speaks to the polymicrobial nature of periodontal disease. Information on the timing of periodontal disease in this cohort was not captured during the study (e.g. former vs. current), nor exact types of treatment received, which may be responsible for some of these differences as well. Overall, although some of the more usual suspects involved in periodontitis were not detected (e.g. *Porphyromonas gingivalis*, *Tannerella forsythesis*, *Treponema denticola*), the shift to a higher abundance of Gram-negative anaerobes is clearly in agreement with previous studies, and no additional dysbiotic interactions between periodontitis and cancer-linked microbes were detected.

Using a carefully controlled cohort, we were able to draw robust conclusions about associations between the oral microbiome and several disease states. By accounting for technical considerations such as sequencing batch effects and self-reporting limitations (e.g. for tobacco and alcohol consumption) during our analysis, we were able to demonstrate that the variances explained by the clinical factors are larger than those explained by batch effect. Thus, the relationships we observed are significant. Although the study design here permitted extensive testing of microbial dysbioses associated with oral cancer and its risk factors, as well as limited functional testing, much work remains in the analysis of the oral cancer microbiome. Causal factors and molecular mechanisms must be identified, with shotgun metagenomics (and/or metatranscriptomics) an obvious potential culture-independent follow-up. In addition to leveraging advances in sequencing methods, future studies should consider environmental factors known to affect the microbiome more broadly (e.g. diet), which we were not able to assess. Ultimately, studies of the microbiome will combine greater molecular detail with larger population sizes and longitudinal monitoring to improve early detection and mitigation of microbial factors in oral cancer.

## Methods

### Participants

Eligible case subjects were identified from among consecutive patients diagnosed with oral cancer at the outpatient otolaryngology clinic of the Ohio State University Comprehensive Cancer Center (Columbus, OH) from July 2011 through May 2013. Patients were eligible for inclusion into the study if they were older than 17 years and were newly diagnosed with a histologically confirmed squamous cell carcinoma of the oral cavity or oropharynx. Anatomic site of origin was determined by a physical examination performed by the treating head and neck surgeon. Individuals with a prior history of head and neck cancer were ineligible.

Eligible controls included patients older than 17 years with no history of cancer who were evaluated as an outpatient for any benign condition between August 2011 and August 2013 at the same otolaryngology service where cases were enrolled. This control population was considered to be representative of the referral population from which the case subjects were identified. After enrollment of a case subject, eligible control subjects in the same gender and age (5-year intervals) categories were invited to participate until two control subjects were individually matched to each case. The study protocol was approved by the Institutional Review Board of the Ohio State University and written informed consent was obtained from all study participants. All methods were performed in accordance with the relevant guidelines and regulations.

Oral rinse samples were collected from case subjects before the initiation of cancer therapy and from control subjects at enrollment. Oral rinse samples were collected by use of a 30-second rinse and gargle with either Scope^TM^ mouthwash or saline.

Detailed demographic and behavioral information was collected from all cases and controls using a computer-assisted interview programmed on a touch screen computer (Apple iPad). Domains included demographics, lifetime measures of tobacco, alcohol and marijuana use and medical and dental history. A “never” user of tobacco was defined as an individual who reported never having used any form of tobacco, including cigarettes, pipe, cigar, chewing tobacco or snuff. A current tobacco user was defined as reporting current use of one or more cigarettes per day or cigar, pipe or smokeless tobacco once or more a week. A “regular smoker” was defined as someone who ever smoked cigarettes daily for one month or more, or who used cigars, pipe or chewing tobacco at least once a week for six months or more. A “former user” is someone who reported they no longer did so at this interval when responding to the survey, making the minimal time frame since cessation one month for cigarette smokers and six months for other tobacco users. The median number of cigarettes per day among current smokers was 20 (IQR 12–27).

A “never” user of alcohol was defined as an individual who reported never having had a drink containing alcohol. A “current” alcohol user was defined as an individual who reported current consumption of one or more drinks per month. One “drink” of beer was defined as 12 ounces, wine as 5 ounces, and liquor as 1.5 ounces. The median number of drinks per week among current drinkers was 8 (IQR 2–20). An individual with a history of periodontitis was defined as an individual who reported having been told by a dental health provider that they had periodontitis. Current tooth status was self-reported in the specified categories.

An HPV-positive tumor was defined as a tumor positive for high-risk HPV E6 or E7 mRNA expression as previously described^84^. Briefly, samples were considered evaluable if DNA (ERV3) and mRNA (RPLPO, after reverse transcription) control templates could be amplified by qPCR and RT-PCR, respectively, after purification from formalin-fixed and paraffin-embedded tumor specimens. Tumor HPV status was determined by detection of HPV DNA of 15-high-risk types by consensus SPF10 primer system PCR amplification followed by reverse line blot hybridization (Inno-LiPa assay, Innogenetics). HPV DNA-positive samples were examined for the presence of E6 E7 mRNA expression of the corresponding HPV type by type-specific RT-PCR, and samples were considered positive if above the lower limit of assay reproducibility^84^.

### DNA extraction

Oral rinse samples were immediately placed at 4 °C and processed as previously described^2^. DNA was purified on a robotic, magnetic-bead based platform (QIAsymphony SP, Qiagen Inc.) using the QIAsymphony virus/bacteria Midi Kit and stored at −80 °C until further analysis.

### 16S rRNA gene sequencing

The 16S gene library consisted of Illumina MiSeq sequences targeting the V4 variable region. Detailed protocols used for 16S amplification and sequencing are as described before^85^. In brief, genomic DNA was subjected to 16S amplification using primers incorporating the Illumina adapters and a sample-specific barcode sequence, allowing directional sequencing covering variable region V4 (Primers: 515 F [GTGCCAGCMGCCGCGGTAA] and 806 R [GGACTACHVGGGTWTCTAAT]). PCR mixtures contained 10 μl of diluted template (1:50), 10 μl of HotMasterMix with the HotMaster Taq DNA Polymerase (5 Prime), and 5 μl of primer mix (2 μM of each primer). The cycling conditions consisted of an initial denaturation of 94 °C for 3 min, followed by 30 cycles of denaturation at 94 °C for 45 sec, annealing at 50 °C for 60 sec, extension at 72 °C for 5 min, and a final extension at 72 °C for 10 min. Amplicons were quantified on the Caliper LabChipGX (PerkinElmer, Waltham, MA), pooled in equimolar concentrations, size selected (375–425 bp) on the Pippin Prep (Sage Sciences, Beverly, MA) to reduce non-specific amplification products from host DNA, and a final library sizing and quantification was performed on an Agilent Bioanalyzer 2100 using DNA 1000 chips (Agilent Technologies, Santa Clara, CA). Sequencing was performed on the Illumina MiSeq v2 platform, according to the manufacturer’s specifications with addition of 5% PhiX, and generating paired-end reads of 150b in length in each direction. The overlapping paired-end reads were stitched together (approximately 97 bp overlap), and size selected to reduce non-specific amplification products from host DNA (22–275 bp).

To mitigate differences in detected microbial abundances arising for technical reasons among our three sequencing batches, we included sequencing phase as a covariate in all analyses. 124 microbial clades were significantly associated with batch identifier after adjusting for other covariates (case/control status, tooth status, etc.) and for multiple comparisons. Adjusting for this batch effect errs on the side of false negatives rather than false positives, but the loss in the power governs the type I error rate, implying that remaining non-batch associations are reliable.

### Operational taxonomy unit (OTU) picking and taxonomy assignment

We used QIIME (version 1.6.0)^86^ to perform OTU picking and taxonomy assignment as specified by PICRUSt^87^ (Supplementary Dataset 1). Specifically, we clustered the sequences from each sample into OTUs at 97% identity using UCLUST^88^ and matched reference sequences against Greengenes 13.5^89^. The resulting OTU tables were checked for mislabeling^90^ and contamination^91^, yielding mean sequence depth of 29,914 reads/sample. We finally applied PICRUST^87^ to predict gene family abundances from our 16S rRNA gene data (Supplementary Dataset 2, summary quality control statistics in Supplementary Dataset 9).

### Metabolic pathway reconstruction

Inferred per-community gene abundances were subsequently reconstructed into microbial small metabolic modules, whose relative abundances were computed using HUMAnN^92,93^. We grouped KEGG orthologues into modules represented as gene sets and determined whether a module is present based on KEGG’s conjunctive normal form logic. Next, we computed each pathway’s relative abundance as a smoothed average over all genes, taking outliers and gap filling into account, which resulted in the relative abundances of modules within each sample. These sample-by-module relative abundances were subsequently treated in the same manner as sample-by-clade microbial abundances. We calculated nearest sequenced taxon index (NSTI) values [80] for all samples from this study, which ranged from a greatest distance of ~0.07 through a minimum of ~0.01 and a mean of ~0.03, suggesting a Spearman correlation with underlying metagenomes between 0.7–0.9, well within the range of biological confidence (Supplementary Fig. 2).

### Significant associations of microbial clades and pathways with sample metadata

OTU tables and reconstructed pathway abundance data were first processed for quality control. Clinical metadata features were not used if more than 10% of the data were missing, or when they did not vary in value over the available samples. Features (i.e. clades or pathways) of very low abundance (<0.001 in ≥90% of samples) as well as feature outliers (>3 × interquartile range in either direction) were removed as described previously^92^. After processing, 363 samples passed quality control for clade and functional abundance analyses.

Clades and functions were tested for statistically significant associations with clinical metadata of interest using a multivariate analysis^92,93^. Here, the relative abundance of each clade was normalized with a variance-stabilizing arcsine square-root transformation and linked with the chosen covariates using a linear model, while the model selection for sparse data was performed per feature using boosting. A multivariate linear model (MaAsLin, http://huttenhower.sph.harvard.edu/maaslin) associating all available metadata (including sequencing batch) with each clade was boosted independently, and any metadata selected in at least 1% of these iterations was tested for significance in a standard generalized linear model. Within each metadatum/feature association, multiple comparisons over factor levels were independently adjusted using a Bonferonni correction; multiple hypothesis tests over all features and metadata associations were adjusted on the Bonferonni corrected p-values to produce a final Benjamini and Hochberg false discovery rate (FDR). Unless otherwise indicated, significant association was considered below a FDR *q*-value of 0.25.

Features included in the multivariate linear model are listed in Supplementary Dataset 3 and comprise case/control status, tooth loss status, tumor HPV status (for cases) by qPCR/RT-PCR/both, periodontal disease, diabetes status, cigarette and tobacco usage, alcohol consumption, microbial DNA concentration, whether a subject was born in the United States, race/ethnicity, age, and gender. All raw sequencing data are available from the SRA at BioProject number PRJNA321193.

## Electronic supplementary material

Supplementary Information Appendix

Dataset 1

Dataset 2

Dataset 3

Dataset 4

Dataset 5

Dataset 6

Dataset 7

Dataset 8

Dataset 9
