# Supplementary material for: Alterations in oral bacterial communities are associated with risk factors for oral and oropharyngeal cancer
Source: Sci Rep. 2017 Dec 15;7:17686. doi: 10.1038/s41598-017-17795-z (PMC5732161; doi:10.1038/s41598-017-17795-z)
Supplement: Supplementary file 1 — Supplementary Information Appendix [file 41598_2017_17795_MOESM1_ESM.pdf]

Supplementary Information for:

Alterations in oral bacterial communities are associated with risk factors for oral and oropharyngeal cancer

Daniela Börnigen*[1,2,7], Boyu Ren*[1,2], Robert Pickard[3], Jingfeng Li[3], Enver Ozer[3], Erica M. Hartmann[4], Weihong Xiao[3], Timothy Tickle[2], Jennifer Rider[5], Dirk Gevers[2], Eric A. Franzosa[1,2], Mary Ellen Davey[6], Maura L. Gillison*[3], Curtis Huttenhower*[1,2]

[1] Department of Biostatistics, Harvard T.H. Chan School of Public Health, Harvard University, Boston, MA 02115, USA

[2] The Broad Institute of MIT and Harvard, Cambridge, MA 02115, USA

[3] The Ohio State University Comprehensive Cancer Center, Columbus, OH 43202, USA

[4] Biology and the Built Environment Center and Institute of Ecology and Evolution, University of Oregon, Eugene, OR 97403, USA. Now at Department of Civil and Environmental Engineering, Northwestern University, Evanston, IL 60208, USA

[5] Department of Epidemiology, Harvard T.H. Chan School of Public Health, Harvard University, Boston, MA 02115, USA

[6] Department of Oral Biology, College of Dentistry, University of Florida, Gainesville, FL 32610, USA

[7] Current address: University Heart Center Hamburg-Eppendorf, Clinic for General and Interventional Cardiology, Hamburg, Germany; German Center for Cardiovascular Research (DZHK), Hamburg/Lübeck/Kiel Partner Site, Hamburg, Germany

Supplementary Figures

Supplementary Figure 1: Alpha and beta diversities stratified by major covariates. Distributions of (**a**) alpha diversities (inverse Simpson's) and (**b**) beta diversities (weighted UniFrac) as stratified by levels of four major study covariates: tobacco usage, alcohol consumption, periodontal health, and HPV positivity. Stars indicate significance at *p*<0.05 by Wilcoxon rank-sum tests. (See **Table 1** and **Supplementary Datasets 4** and **5**).

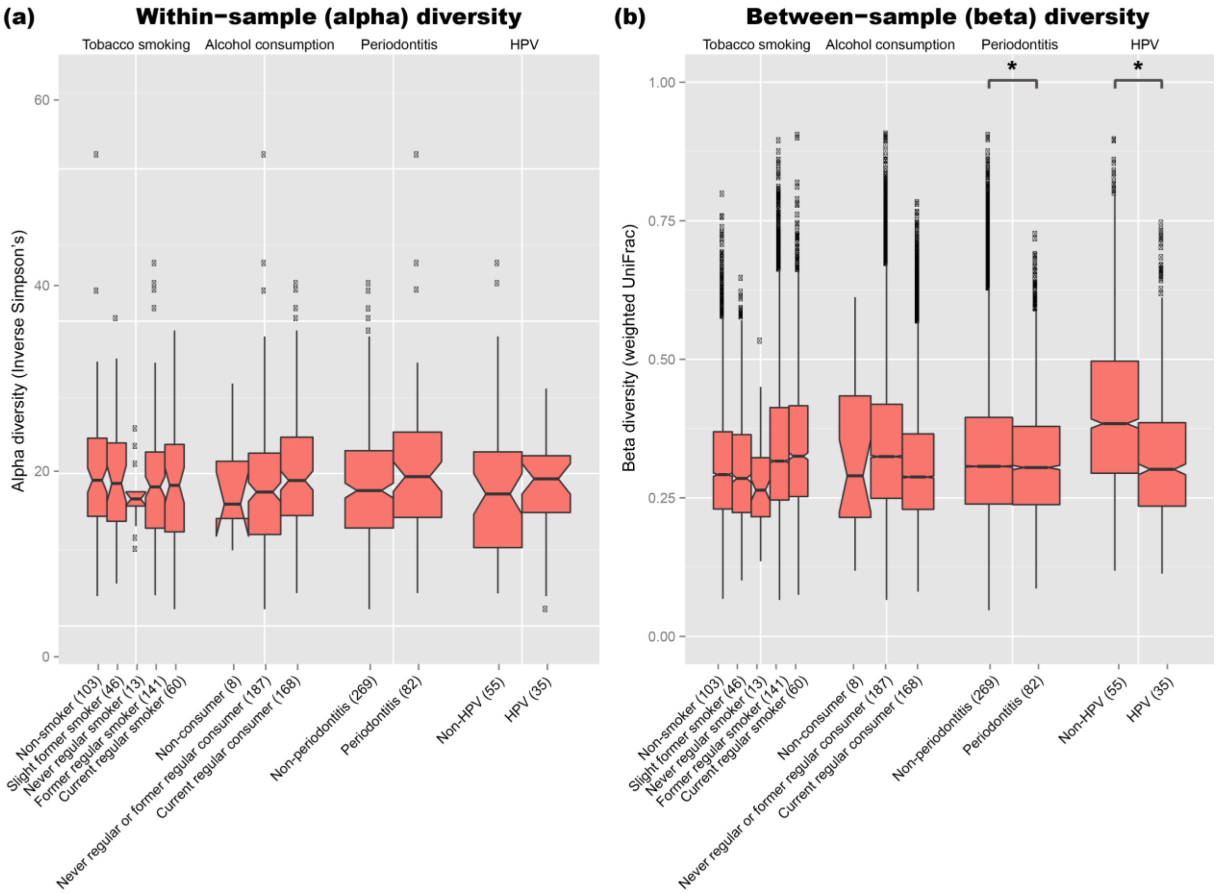

Supplementary Figure 2: Nearest Sequenced Taxon Index values from PICRUSt functional inference. Distribution indicates NSTI (Nearest Sequenced Taxon Index) values as calculated by PICRUSt during inference of approximate functional profiles across all samples. As described in the PICRUSt manuscript, values <0.05 generally correspond to expected Spearman correlations >0.8 between inferred and true functional profiles.

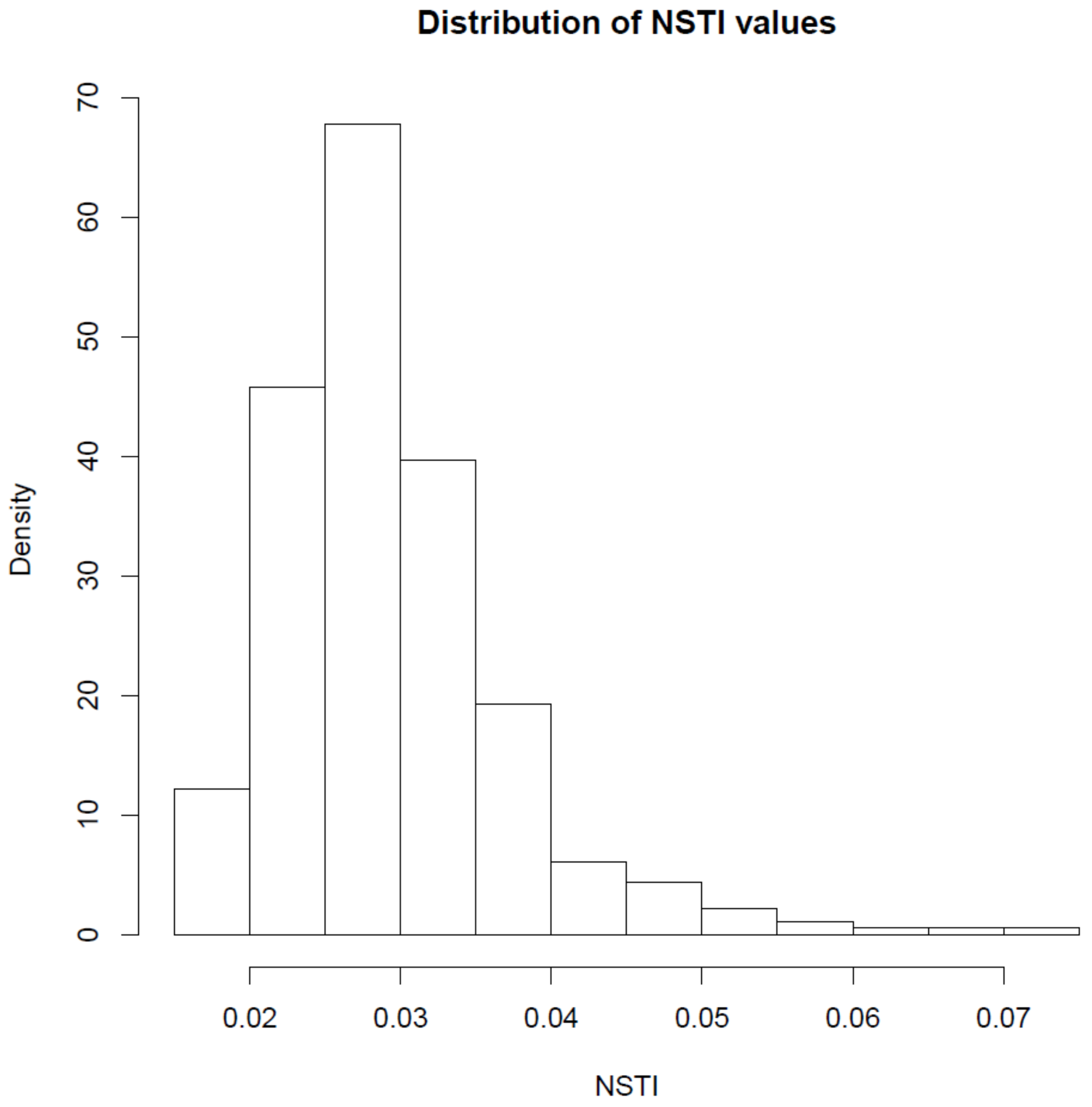

Descriptions of Supplementary Datasets

Supplementary Dataset 1: Taxonomic profiles (OTU table) from all samples.

Supplementary Dataset 2: Functional inferences (PICRUSt table) from all samples.

Supplementary Dataset 3: Samples annotated with study metadata covariates.

Supplementary Dataset 4: Significant microbial abundances as detected for cancer vs. healthy, healthy tooth status vs. no natural teeth left, smokers vs. non-smokers, HPV infected cancer patients vs. non-HPV infected cancer patients, and periodontitis vs. healthy.

Supplementary Dataset 5: Significant modules and pathways as detected for cancer vs. healthy, healthy tooth status vs. no natural teeth left, smokers vs. non-smokers, HPV infected cancer patients vs. non-HPV infected cancer patients, and periodontitis vs. healthy.

Supplementary Dataset 6: Variance explained in taxonomic profiles by major covariates.

Supplementary Dataset 7: Variance explained in inferred functional profiles by major covariates.

Supplementary Dataset 8: Effect sizes (coefficient values) for major covariates with respect to taxonomic and inferred functional features.

Supplementary Dataset 9: Read-level count and taxonomic assignment summary statistics.
